# Urinary Peptidomics and Pulse Wave Velocity: The African-PREDICT Study

**DOI:** 10.1021/acs.jproteome.3c00347

**Published:** 2023-09-09

**Authors:** Dalene de Beer, Catharina MC Mels, Aletta E Schutte, Christian Delles, Sheon Mary, William Mullen, Harald Mischak, Ruan Kruger

**Affiliations:** † Hypertension in Africa Research Team (HART),North-West University (Potchefstroom Campus), Potchefstroom 2531, South Africa; ‡ MRC Research Unit for Hypertension and Cardiovascular Disease, North-West University, Potchefstroom 2520, South Africa; § School of Population Health, The George Institute for Global Health, University of New South Wales, Sydney, NSW 2042, Australia; ∥ School of Cardiovascular and Metabolic Health, University of Glasgow, Glasgow G12 8QQ, U.K.; ⊥ Mosaiques Diagnostics GmbH, Hannover D-30659, Germany

**Keywords:** arterial stiffness, early vascular aging, vascular extracellular matrix, pathway analysis, collagen type I

## Abstract

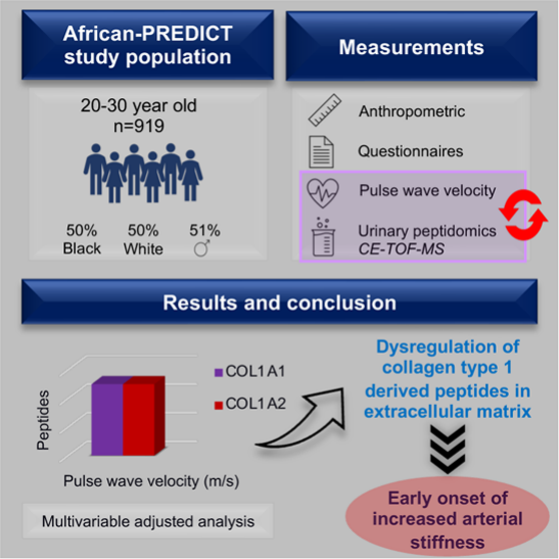

Increased arterial stiffness is related to early vascular aging and is an independent predictor for cardiovascular disease and mortality. Molecular mechanisms underlying increased arterial stiffness are largely unexplored, especially at the proteome level. We aimed to explore the relationship between pulse wave velocity and urinary proteomics. We included 919 apparently healthy (no chronic illnesses) Black and White men and women (equally distributed) between 20 and 30 years from the African-PREDICT study. Capillary electrophoresis time-of-flight mass spectrometry was used to analyze the urinary proteome. We measured the carotid-femoral pulse wave velocity to estimate arterial stiffness. In the total group, pulse wave velocity correlated positively with collagen-derived peptides including collagen types I, II, III, IV, V, and IX and inversely with collagen type XI (adjusted for mean arterial pressure). Regarding noncollagen-derived peptides, pulse wave velocity positively correlated with polymeric immunoglobulin receptor peptides (*n* = 2) (all *q*-value ≤0.05). In multivariable adjusted analyses, pulse wave velocity associated positively and independently with seven urinary peptides (collagen type I, *n* = 5) (all *p*-value ≤0.05). We found significant positive and independent associations between pulse wave velocity and the collagen type I-derived peptides, suggesting that dysregulation of collagen type I in the extracellular matrix scaffold could lead to early onset of increased arterial stiffness.

## Highlights

- Pulse wave velocity associated positively with collagen type I-derived peptides in a young and healthy population.
- Collagen type I is one of the main collagen proteins found in the vascular ECM and is responsible for the stability and function of blood vessels.
- The dysregulation of collagen types I and III turnover may lead to increased arterial stiffness.

## Introduction

Aortic pulse wave velocity (PWV) is the gold standard measurement of arterial stiffness,^1^ and increased arterial stiffness, especially at younger ages, may reflect early vascular aging.^2^ Pulse wave velocity is a strong and independent predictor for increased cardiovascular disease (CVD) risk and all-cause mortality,^3,4^ even at young ages.^3,5^ Increased arterial stiffness with aging may be accelerated by factors such as oxidative stress, inflammation, endothelial dysfunction, and hemodynamic forces^6,7^ and may reflect subclinical organ damage.^8,9^ The molecular mechanisms underlying these preclinical changes are largely unexplored but remain important from a personalized medicine perspective. The use of omics-based biomarkers and, in particular, urinary proteomics may be useful in this regard since it provides more insight into the structure and function of a biological system than genomics.^10,11^

Proteomics is a rapidly growing field in “omics” that provides the ability to study the function, structure, and interactions of proteins at a certain point in time.^12^ Various proteomics studies have successfully identified biomarkers unique to diseases such as coronary artery disease,^13,14^ chronic kidney disease,^15,16^ and heart failure.^17^ Previous proteomics studies focusing on arterial stiffness were limited to very small sample sizes,^18^ which were performed in older and diseased populations^19^ or in plasma samples from unhealthy patients (some presenting with obesity, diabetes, hypertension, peripheral atherosclerotic disease, etc.) with low and high PWV (mean age 65.5 years old)^20^ or arterial tissue samples in young and healthy adults (18–26 years old).^18^ To the best of our knowledge, no urinary proteomics study has focused on the early molecular phenotype of arterial stiffness in young adults.

In a recent study on urinary proteomics and early CVD risk,^21^ we compared the urinary peptide abundances between low, medium, and high CVD risk groups and found that collagen type I and III- derived peptides were lower in the high compared to the low CVD risk group, suggesting potential early alterations in the vascular extracellular matrix.^21^ Therefore, we aimed to determine whether a measure of large artery stiffness (PWV) is associated with a urinary proteomics profile in young adults, with a specific focus on vascular-specific extracellular matrix proteins such as collagen type I and III.

## Methods

### Study Population and Organizational Procedures

This study forms part of The African Prospective Study on the Early Detection and Identification of Cardiovascular Disease and Hypertension (African-PREDICT). The African-PREDICT study has a longitudinal design, aiming to characterize the development of hypertension over a follow-up period of 10 years. Baseline data included 1202 healthy adults (aged 20–30 years).^22^ Participants were recruited from the North-West Province of South Africa. If the participants met the inclusion criteria of the screening phase (self-reported Black or White ethnicity, aged 20–30 years, men or women with no self-reported chronic illness or use of chronic medication, HIV uninfected and clinic normotensive (office brachial blood pressure <140/90 mmHg)), they were invited to join the research phase of the study. For this study, we included 964 participants with complete urinary peptidomics data. All participants with incomplete PWV data (*n* = 45) were excluded, leaving a total group of *n* = 919. Participants signed a written informed consent form to participate in both the screening and research phase of the study. Both the African-PREDICT study (NWU-00001-12-A1) and this substudy (NWU-00495-19-A1) were approved and registered by the Health Research Ethics Committee of the North-West University (ClinicalTrial.gov identifier: NCT03292094).

### Clinical Measurements

A detailed description of questionnaires, physical activity monitoring, and anthropometric, cardiovascular, and biochemical measurements were previously described.^21^

Briefly, a general health and demographic questionnaire was completed for each participant. The Kuppuswamy’s Socioeconomic status scale was used to calculate each participant’s socio-economic score for a South African environment.^23^ ActiHeart physical activity monitors (CamNtech Ltd., London, UK) were used to measure energy expenditure and to record the participant’s heart rate variability. The International Society for the Advancement of Kinanthropometry^24^ guidelines were followed to perform anthropometric measurements with the use of the following apparatuses: a SECA 213 Portable Stadiometer (SECA, Hamburg, Germany) to measure height, a SECA 813 Electronic Scales with a weighing capacity up to 200 kg (SECA, Hamburg, Germany) to measure weight (kg), and a Lufkin Steel Anthropometric Tape (W606PM; Lufkin, Apex, USA) to measure waist circumference (cm). Body mass index (BMI) was calculated by dividing the weight (kg) by height (m^2^). A Dinamap Procare 100 Vital Signs Monitor (GE Mediacal Systems, Milwaukee, USA) with an appropriate sized cuff was used to calculate systolic and diastolic blood pressure as well as heart rate. Carotid-femoral pulse wave velocity was measured noninvasively with the use of a SphygmoCor XCEL device (AtCor Medical Pty. Ltd., Sydney, Australia). Participants were in a supine position and relaxed before the measurements took place. Pulse wave velocity was performed by placing a brachial cuff on the right upper-arm and measured in duplicate along the descending thoraco-abdominal aorta using a foot-to-foot velocity method.

Regarding biochemical analysis, blood sampling and an early morning spot urine sample were taken after the participant fasted for a period of 8 h. Basic biochemical measurements included serum total cholesterol, low-density lipoprotein cholesterol (LDL-c), high-density lipoprotein cholesterol (HDL-c), triglycerides, glucose, gamma-glutamyl transferase (GGT), glycated hemoglobin (HbA1c), C-reactive protein (CRP) (Cobas Integra 400 plus, Roche, Basel, Switzerland), and serum cotinine (Immulite, Siemens, Erlangen, Germany). With regard to urinary peptidomics analyses, capillary electrophoresis time-of-flight mass spectrometry (CE-TOF-MS) was performed using a P/ACE MDQ capillary electrophoresis system (Beckman Coulter, Fullerton, USA) coupled with a microTOF mass spectrometer (Bruker Daltonic, Bremen, Germany) as previously described.^25^ A detailed description on the biochemical analysis, sample preparation, and identification of urinary peptidomics was published previously.^21^

### Statistical Analysis

We used R version 3.6.0 software (R Foundation for Statistical Computing, Vienna),^26^ IBM SPSS Statistics version 25 software (IBM Corporation; Armonk, New York, USA), and G*Power version 3.1.9.3 software (Faul, Erdfelder, Lang, & Buchner, 2007)^27^ to perform statistical analyses.

QQ-plots were used to test the normality of biochemical variables, and skewed data (lipids, GGT, HbA1c, CRP and cotinine) were logarithmically transformed. We performed descriptive analyses to summarize the characteristics of the total group (*n* = 919) (Table 1).

**Table 1:** Characteristics of the African-PREDICT Population^a^

	total group (*n* = 919)
age (years)	24.4 ± 3.12
ethnicity, Black *n* (%) and White *n* (%)	457 (49.7); 462 (50.3)
sex, male *n* (%)	467 (51)
anthropometry	
height (m)	169 ± 9.58
weight (kg)	71.0 ± 16.6
waist circumference (cm)	80.1 ± 12.1
body mass index (kg/m^2^)	24.9 ± 5.28
cardiovascular measurements	
SBP (mmHg)	118 ± 11.8
DBP (mmHg)	79 ± 7.91
pulse wave velocity (m/s)	6.28 (5.10; 7.85)
heart rate (beats/min)	64 ± 10.04
biochemical analysis	
total cholesterol (mmol/L)	3.44 (1.95; 5.76)
LDL cholesterol (mmol/L)	2.17 (1.02; 4.17)
HDL cholesterol (mmol/L)	1.04 (0.55; 1.87)
triglycerides (mmol/L)	0.70 (0.30; 1.82)
glucose (mmol/L)	3.85 (2.38; 5.54)
cotinine (ng/mL)	3.64 (1.00; 327)
γ-glutamyl transferase (U/l)	17.6 (5.80; 54.3)
HbA1c (%)	5.29 (4.77; 5.81)
C-reactive protein (mg/L)	0.83 (0.07:9.25)
lifestyle	
self-reported smoking, *n* (%)	223 (24)
self-reported alcohol use, *n* (%)	495 (54)
physical activity (kcal/kg/day)	224 ± 364
socio-economic score	20.3 ± 6.10

a Values are arithmetic mean and standard deviation, geometric mean (5th and 95th percentile). Abbreviations: DBP: diastolic blood pressure, SBP: systolic blood pressure, LDL: low-density lipoprotein, HDL: high-density lipoprotein, HbA1c: glycated hemoglobin.

With regard to peptide data, all peptides with >45% missing or undetectable values were excluded for further data analyses. The remaining peptides were logarithmic (log_2_) transformed to obtain comparable intensity ranges. In this study, we further explored whether PWV correlated with the peptides previously identified as being potentially associated with cardiovascular disease risk (*n* = 147)^21^ by performing partial regression analysis (adjusting for mean arterial pressure (MAP)) and multivariable adjusted regression analyses (backward elimination regressions) to determine independent relationships between PWV and the urinary peptides. For partial regression analysis, we also adjusted for multiple comparisons (Benjamini-Hochberg) (*q*-value ≤0.05). The covariates considered for entry in the multiple regression models were age, sex, ethnicity, MAP, heart rate, BMI, physical activity (kcal/kg/day), LDL-c, GGT, cotinine, HbA1c, and CRP.

### Pathway Analysis

We used STRING database v11.5^28^ to perform pathway analysis and explore molecular functions of the proteins that associated significantly with PWV after multivariable adjustments. Gene Ontology (GO) enrichment analysis is determined by a hypergeometric test followed by a false discovery rate (FDR) distribution. The pathways were sorted according to an FDR ≤ 0.05, which describes how likely the pathway enrichment is by chance.

## Results

The general characteristics of the study population (*n* = 919) are described in Table 1. The mean age for this group was 24.4 years, with a similar distribution of sex (51% men, 49.0% women) and ethnicity (49.7% Black, 50.3% White).

### Partial and Multivariable Regression Analysis

**Figure 1:**
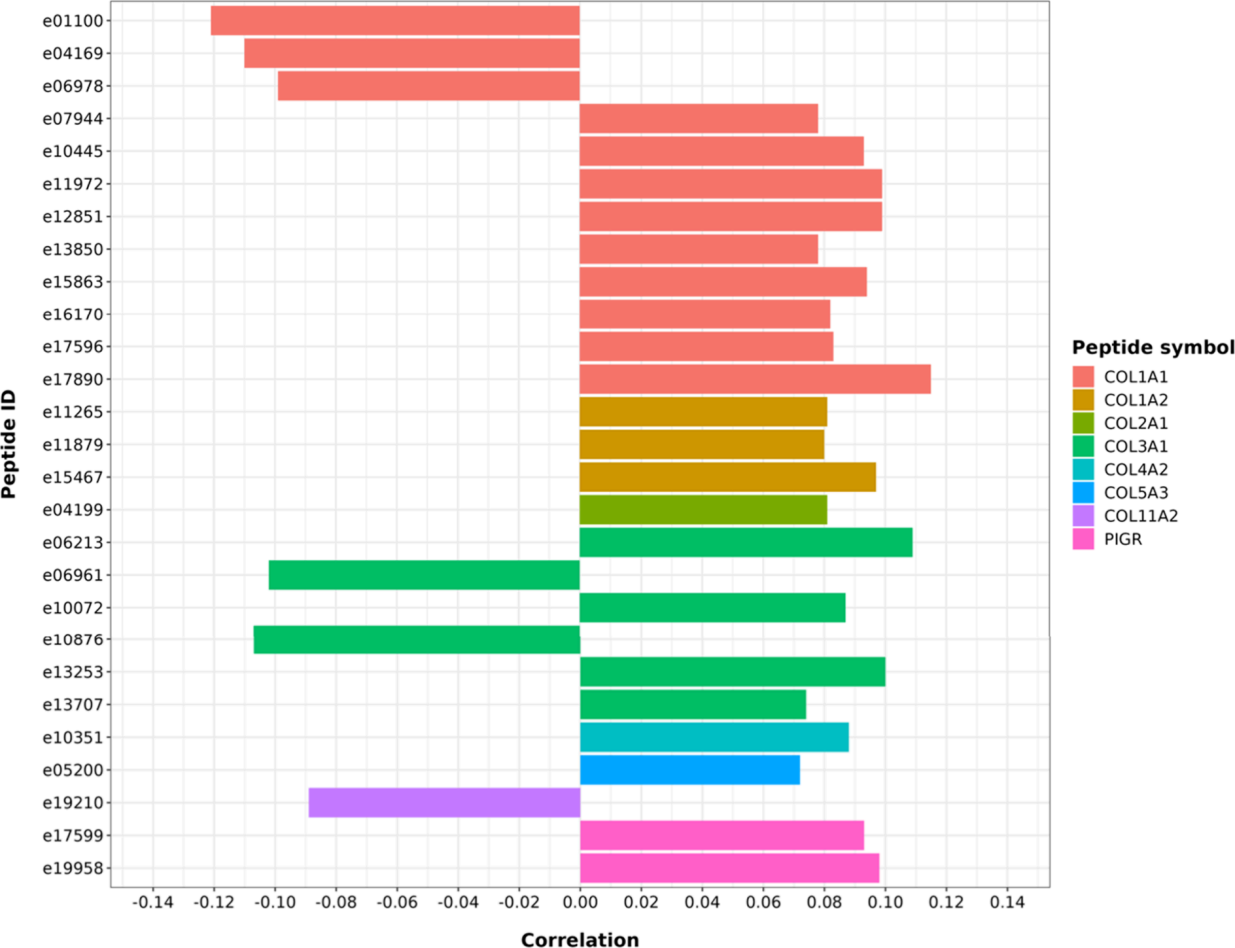
Regression analysis between pulse wave velocity and urinary peptides in the total group (*n* = 919), adjusted for mean arterial pressure. The bars represent the regression coefficient ‘*r*’, all *q*-value <0.05.

In the total group, after adjustment for mean arterial pressure, pulse wave velocity correlated positively with several collagen alpha-1(I) (COL1A1)-derived peptides (represented by 12 peptides) and negatively with peptides e01100, e04169, and e06978 (all *q*-value ≤0.05). Pulse wave velocity also correlated positively with collagen alpha-2(I) (COL1A2)-derived peptides (represented by three peptides) (all *q*-value ≤0.039) as well as collagen alpha-3(I) (COL3A1)-derived peptides (represented by six peptides) and negatively with peptides e06961 and e10876 (all *q*-value ≤0.036) (Figure 1, Supplementary Table 1).

Pulse wave velocity correlated positively with collagen alpha-1(II) (COL2A1) (represented by one peptide), collagen alpha-2(IV) (COL4A2) (represented by one peptide), and collagen alpha3(V) (CO5A3) (represented by one peptide) and inversely with collagen alpha-2(XI) (COL11A2) (represented by one peptide) (all *q*-value ≤0.05). Regarding noncollagen peptides, PWV positively correlated with a polymeric immunoglobulin receptor (PIGR) (represented by two peptides) (all *q*-value ≤0.032) (Figure 1, Supplementary Table 1).

**Figure 2:**
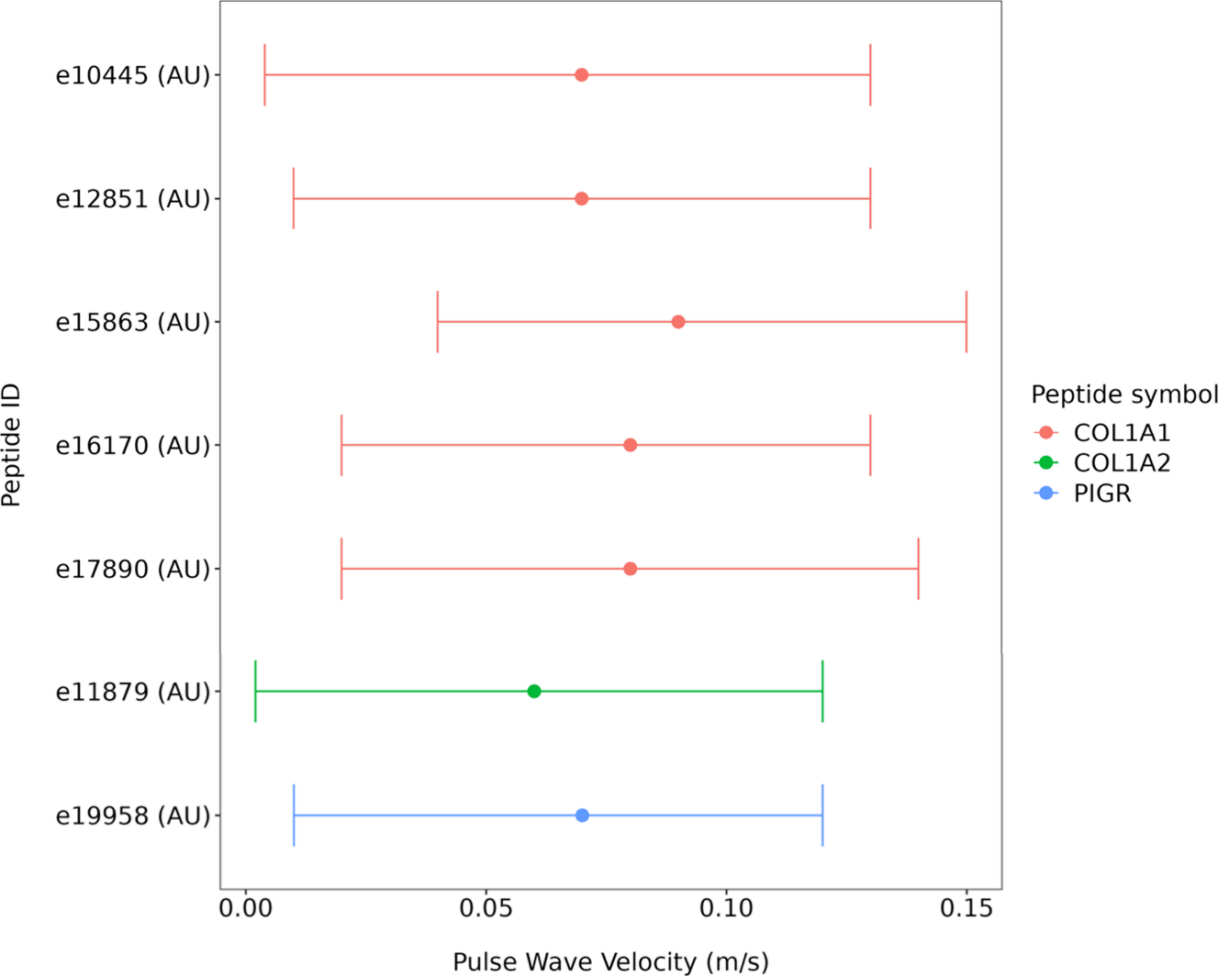
Multivariable adjusted analysis between pulse wave velocity and the identified urinary peptides (*n* = 7), adjusted for sex, age, mean arterial pressure, heart rate, body mass index, γ-glutamyl transferase, glycated hemoglobin, C-reactive protein, cotinine, low-density lipoprotein, and physical activity, all *p* < 0.05.

In multivariable adjusted regression analysis (Figure 2, Supplementary Table 2), PWV associated positively and independently with COL1A1 (represented by five peptides) and COL1A2 (represented by one peptide) as well as with PIGR (represented by one peptide) (all *p*-value ≤0.044).

### Pathway Analysis

After performing pathway analysis with the peptides that associated with PWV in multivariable adjusted analysis (*n* = 3 types of peptides) (COL1A1, COL1A2 and PIGR), we identified two pathways for molecular functions (Supplementary Table 3). The functional annotations of the identified urinary peptides revealed that the main Gene Ontology (GO) terms of molecular functions wherein the majority of urinary peptides overlapped (COL1A1 and COL1A2) were (i) extracellular matrix structural constituent conferring tensile strength and (ii) platelet-derived growth factor binding. Also, in STRING database (Supplementary Table 3), Reactome pathways involved in the vascular ECM included (i) anchoring fibril formation, (ii) platelet adhesion to exposed collagen, (iii) cross-linking of collagen fibrils, (iv) platelet aggregation (plug formation), (v) collagen chain trimerization, (vi) collagen degradation, (vii) ECM proteoglycans, (viii) integrin cell surface interactions, and (ix) cell surface interactions at the vascular wall.

## Discussion

We performed detailed urinary proteomic analyses in young and apparently healthy adults to determine the relationships between the pulse wave velocity and urinary peptides specific to the vascular extracellular matrix (ECM). We found that the gold standard measure of arterial stiffness, PWV, was associated significantly and independently with seven urinary peptides. The majority were collagen type 1-derived peptides. Moreover, in STRING analysis, we also identified molecular functions and Reactome pathways associated with the vascular ECM. We suggest that if these pathways are dysregulated, it may lead to the earlier development of arterial stiffness and should therefore be closely monitored in high risk individuals from a young age.

The urinary peptides associated with PWV in this study are similar to previous proteomics studies in the field of arterial stiffness, such as collagen types I, II, and III.^18,19^ However, proteomics studies focusing on arterial stiffness are limited and it remains challenging to compare results across different age groups and various sample matrices in either healthy or diseased populations.^18−20^ Recently, a study developed a PWV urinary proteomics score in the Flemish Study on Environment, Genes, and Health Outcomes population (mean age 50 years old) and found it to prospectively associate with all-cause mortality and cardiovascular outcome over a period of 9.2 years.^19^ In the latter study, the majority of the peptides in the urinary proteomic profile consisted of collagen type I and III fragments and were mostly associated negatively with PWV. This is in contrast with our findings of positive associations between PWV and urinary peptides. We suggest that with advancing age, the impact on collagen generation may change with increasing collagen accumulation, increased nonenzymatic glycation and collagen cross-linking,^29^ and consequently less collagen degradation. Nonetheless, our findings add to this since we identified collagen type I fragments to be associated with PWV in a large young and healthy population without end-organ damage, which may suggest that the dysregulation of collagen type I turnover may lead to increased arterial stiffness in the setting of early vascular aging.

## Collagen-Derived Peptides

Collagen type I and III are the main collagen proteins in the vascular ECM with collagen type I being the most prominent of collagen fibrils.^30^ Collagens type I and III are found in the tunica adventitia of the arterial wall and play a pivotal role in the mechanical and tensile strength and contractility of arteries,^30^ whereas elastin, found in the media of the arterial wall, is responsible for vascular elasticity.^31,32^ As such, an unbalanced vascular ECM turnover will stimulate vascular remodeling and may consequently induce early vascular aging and increased arterial stiffness.

## Potential Mechanisms That May Lead to Increased Arterial Stiffness

In these young and healthy subjects without end-organ damage, there is no evidence of chronic pathological processes on collagen cross-linking. We propose that if inflammation and protease activity increase as seen with increasing arterial stiffness, urinary collagen fragments will also increase, hence the positive associations between PWV and urinary peptides. In pathway analysis, we showed that collagen type I-derived peptides are involved in processes that are related to the vascular ECM, such as platelet aggregation, collagen cross-linking, and degradation. Research has shown that platelets are recruited to inflamed vessels or to the site of injury and can perform pro- or anti-inflammatory reactions, depending on the cause of inflammation.^33^ In addition to the respective peptides, GGT, a marker associated with vascular inflammation,^34^ also contributed significantly to the variance in PWV. With an increase in vascular inflammation, matrix metalloproteinases (MMPs), which are responsible for the degradation of proteins in the ECM, are also up-regulated.^35^ Increased MMPs, such as MMP2 and MMP9, are associated with arterial stiffness, even in younger and apparently healthy subjects.^36,37^ We therefore propose that if vascular inflammation increases, protease activity may also increase, leading to early changes in the vascular ECM, including degraded and fragmented elastin and increased collagen degradation.^38^ This may lead to the up-regulation of collagen biosynthesis^39^ and deposition to counteract the potentially harmful effects of inflammation and MMPs on the vascular ECM structure, which may over time enhance collagen cross-linking and increase arterial stiffness.^40^

In addition to the respective peptides, MAP (as expected) also contributed significantly to the variance in PWV. Since we found positive associations between PWV and collagen type I-derived peptides, we propose that with increased blood pressure, mechanical stress in blood vessels will be elevated.^41,42^ This may lead to the up-regulation of collagen biosynthesis and deposition to maintain vascular ECM homeostasis in the midst of higher mechanical strain exerted on the arterial walls, which may ultimately result in poorly organized nonenzymatically collagen cross-linking,^32^ contributing to increased arterial stiffness. We therefore propose that collagen type I-derived peptides may play a key role in our understanding of structural vascular ECM changes, leading to higher arterial stiffness and early vascular aging. Regarding noncollagen peptides, PWV associated significantly with PIGR; however, more research is needed to explore the possible role between PIGR and arterial stiffness.

## Strengths and Limitations

To the best of our knowledge, this is the first urinary proteomics study to show independent associations between PWV and vascular ECM specific peptides involved in pathways related to extracellular matrix organization in a young and healthy population. Even though PWV is within normal ranges in this study, the impact of PWV on urinary peptides is significant and consistent. This study did not include MMP data, and further investigation is proposed to test our hypotheses with regard to the role of MMPs with urinary peptides in the early changes within the vascular ECM. Data collection for the first follow-up phase of the African-PREDICT study is continuing, which will enable hypothesis testing in a longitudinal setting, to explore the development of arterial stiffness and early vascular aging over time, as well as the predictive value of the identified peptidome in young asymptomatic adults.

## Conclusions

In conclusion, in healthy young adults, we found positive and independent associations between PWV and collagen type-I derived peptides. Our findings likely reflect early mechanisms for early vascular aging associated with the structural integrity and function of the vascular ECM as described by peptides that indicate collagen turnover, high tensile strength under mechanical strain, and cell signaling for vascular remodeling.

## Supporting Information Available

Supplementary Table 1: Partially adjusted linear regression analyses between pulse wave velocity and urinary peptides in the total group; Supplementary Table 2: Multiple regression analysis of pulse wave analysis with identified peptides in the total group; Supplementary Table 3: Interaction network of enriched Gene Ontology (GO) terms and pathway analysis of the identified urinary peptides (PDF)

## Notes

Data sharing is available from the principal investigator of the African-PREDICT study on reasonable request. A data sharing agreement will be set up and submitted to the Health Research Ethics Committee of the North-West University, where data will be shared if approved by the Health Research Ethics Committee.

## Abbreviations

BMI: body mass index
CVD: cardiovascular disease
CE-TOF-MS: capillary electrophoresis time-of-flight mass spectrometry
COL1A1: collagen alpha-1(I)
COL2A1: collagen alpha-1(II)
COL2A1: collagen alpha-1(II)
COL4A1: collagen alpha-1(IV)
COL1A2: collagen alpha-2(I)
COL4A2: collagen alpha-2(IV)
COL11A2: collagen alpha-2(XI
COL3A1: collagen alpha-3(I)
COL9A3: collagen alpha-3(IX)
ECM: extracellular matrix
FDR: false discovery rate
GGT: gamma-glutamyl transferase
GO: Gene Ontology
HbA1c: glycated hemoglobin
HDL-c: high-density lipoprotein cholesterol
LDL-c: low-density lipoprotein cholesterol
MMPs: matrix metalloproteinases
MAP: mean arterial pressure
PWV: pulse wave velocity
African-PREDICT: The African Prospective Study on the Early Detection and Identification of Cardiovascular Disease and Hypertension
kcal: kilocalorie
kg: kilogram
m: meter

## References

[ref1] Van Bortel L. M.; Laurent S.; Boutouyrie P.; Chowienczyk P.; Cruickshank J. K.; De Backer T.; Filipovsky J.; Huybrechts S.; Mattace-Raso F. U.; Protogerou A. D. Expert consensus document on the measurement of aortic stiffness in daily practice using carotid-femoral pulse wave velocity. J. Hypertens. 2012, 30, 445–448. 10.1097/HJH.0b013e32834fa8b0. 22278144

[ref2] Bruno R. M.; Nilsson P. M.; Engström G.; Wadström B. N.; Empana J.-P.; Boutouyrie P.; Laurent S. Early and supernormal vascular aging. Hypertension. 2020, 76, 1616–1624. 10.1161/HYPERTENSIONAHA.120.14971. 32895017

[ref3] Vlachopoulos C.; Aznaouridis K.; Stefanadis C. Prediction of cardiovascular events and all-cause mortality with arterial stiffness: A systematic review and meta-analysis. J. Am. Coll Cardiol. 2010, 55, 1318–1327. 10.1016/j.jacc.2009.10.061. 20338492

[ref4] Bonarjee V. V. S. Arterial stiffness: A prognostic marker in coronary heart disease. Available methods and clinical application. Front. Cardiovasc. Med. 2018, 5, 64 10.3389/fcvm.2018.00064. 29951487 PMC6008540

[ref5] Mikael L. d. R.; Paiva A. M. G. d.; Gomes M. M.; Sousa A. L. L.; Jardim P. C. B. V.; Vitorino P. V. d. O.; Euzébio M. B.; Sousa W. d. M.; Barroso W. K. S. Vascular aging and arterial stiffness. Arq. Bras. Cardiol. 2017, 109, 253–258. 10.5935/abc.20170091. 28678931 PMC5586233

[ref6] Lyle A. N.; Raaz U. Killing me unsoftly: Causes and mechanisms of arterial stiffness. Arterioscler., Thromb., Vasc. Biol. 2017, 37, e1–e11. 10.1161/ATVBAHA.116.308563. 28122777 PMC5308873

[ref7] Lacolley P.; Regnault V.; Laurent S. Mechanisms of arterial stiffening. Arterioscler., Thromb., Vasc. Biol. 2020, 40, 1055–1062. 10.1161/ATVBAHA.119.313129. 32075419

[ref8] Tomiyama H.; Ishizu T.; Kohro T.; Matsumoto C.; Higashi Y.; Takase B.; Suzuki T.; Ueda S.; Yamazaki T.; Furumoto T.; et al. Longitudinal association among endothelial function, arterial stiffness and subclinical organ damage in hypertension. Int. J. Cardiol. 2018, 253, 161–166. 10.1016/j.ijcard.2017.11.022. 29174285

[ref9] Heffernan K.; Spartano N.; Augustine J.; Lefferts W.; Hughes W.; Garay Redmond J.; Martin E.; Gump B.; Kuvin J. Arterial stiffness as a noninvasive tissue biomarker of cardiac target organ damage. Curr. Biomarker Find. 2014, 4, 23–34. 10.2147/CBF.S38738.

[ref10] Holman J. D.; Dasari S.; Tabb D. L. Informatics of protein and posttranslational modification detection via shotgun proteomics. Methods Mol. Biol. 2013, 1002, 167–179. 10.1007/978-1-62703-360-2_14. 23625403 PMC4012295

[ref11] Corlin L.; Liu C.; Lin H.; Leone D.; Yang Q.; Ngo D.; Levy D.; Cupples L. A.; Gerszten R. E.; Larson M. G.; Vasan R. S.; et al. Proteomic signatures of lifestyle risk factors for cardiovascular disease: A cross-sectional analysis of the plasma proteome in the framingham heart study. J. Am. Heart Assoc. 2021, 10, e018020 10.1161/JAHA.120.018020. 33372532 PMC7955453

[ref12] Al-Amrani S.; Al-Jabri Z.; Al-Zaabi A.; Alshekaili J.; Al-Khabori M. Proteomics: Concepts and applications in human medicine. World J. Biol. Chem. 2021, 12, 57–69. 10.4331/wjbc.v12.i5.57. 34630910 PMC8473418

[ref13] Delles C.; Schiffer E.; von zur Muhlen C.; Peter K.; Rossing P.; Parving H. H.; Dymott J. A.; Neisius U.; Zimmerli L. U.; Snell-Bergeon J. K.; Maahs D. M.; Schmieder R. E.; Mischak H.; Dominiczak A. F. Urinary proteomic diagnosis of coronary artery disease: Identification and clinical validation in 623 individuals. J. Hypertens. 2010, 28, 2316–2322. 10.1097/HJH.0b013e32833d81b7. 20811296

[ref14] Zimmerli L. U.; Schiffer E.; Zürbig P.; Good D. M.; Kellmann M.; Mouls L.; Pitt A. R.; Coon J. J.; Schmieder R. E.; Peter K. H.; Mischak H.; Kolch W.; Delles C.; Dominiczak A. F. Urinary proteomic biomarkers in coronary artery disease. Mol. Cell. Proteom. 2008, 7, 290–298. 10.1074/mcp.M700394-MCP200. 17951555

[ref15] Verbeke F.; Siwy J.; Van Biesen W.; Mischak H.; Pletinck A.; Schepers E.; Neirynck N.; Magalhães P.; Pejchinovski M.; Pontillo C. The urinary proteomics classifier chronic kidney disease 273 predicts cardiovascular outcome in patients with chronic kidney disease. Nephrol Dial Transplant. 2021, 36 (5), 811–818. 10.1093/ndt/gfz242. 31837226

[ref16] Øvrehus M. A.; Zürbig P.; Vikse B. E.; Hallan S. I. Urinary proteomics in chronic kidney disease: Diagnosis and risk of progression beyond albuminuria. Clin. Proteom. 2015, 12, 21–21. 10.1186/s12014-015-9092-7. PMC4528848 26257595

[ref17] Zhang Z.; Ravassa S.; Nkuipou-Kenfack E.; Yang W.; Kerr S. M.; Koeck T.; Campbell A.; Kuznetsova T.; Mischak H.; Padmanabhan S.; Dominiczak A. F.; Delles C.; Staessen J. A. Novel urinary peptidomic classifier predicts incident heart failure. J. Am. Heart Assoc. 2017, 6, e005432 10.1161/JAHA.116.005432. 28784649 PMC5586413

[ref18] Lyck Hansen M.; Beck H. C.; Irmukhamedov A.; Jensen P. S.; Olsen M. H.; Rasmussen L. M. Proteome analysis of human arterial tissue discloses associations between the vascular content of small leucine-rich repeat proteoglycans and pulse wave velocity. Arterioscler. Thromb. Vasc. Biol. 2015, 35, 1896–1903. 10.1161/ATVBAHA.114.304706. 26069235

[ref19] Wei D.; Melgarejo J. D.; Thijs L.; Temmerman X.; Vanassche T.; Van Aelst L.; Janssens S.; Staessen J. A.; Verhamme P.; Zhang Z. Urinary proteomic profile of arterial stiffness is associated with mortality and cardiovascular outcomes. J. Am. Heart Assoc. 2022, 11, e024769 10.1161/JAHA.121.024769. 35411793 PMC9238473

[ref20] Pettersson-Pablo P.; Cao Y.; Breimer L. H.; Nilsson T. K.; Hurtig-Wennlöf A. Pulse wave velocity, augmentation index, and carotid intima-media thickness are each associated with different inflammatory protein signatures in young healthy adults: The lifestyle, biomarkers and atherosclerosis study. Atherosclerosis. 2020, 313, 150–155. 10.1016/j.atherosclerosis.2020.09.027. 33059161

[ref21] De Beer D.; Mels C. M. C.; Schutte A. E.; Delles C.; Mary S.; Mullen W.; Mischak H.; Kruger R. A urinary peptidomics approach for early stages of cardiovascular disease risk: The African-PREDICT study. Hypertens Res. 2023, 46 (2), 485–494. 10.1038/s41440-022-01097-7. 36396816

[ref22] Schutte A. E.; Gona P. N.; Delles C.; Uys A. S.; Burger A.; Mels C. M.; Kruger R.; Smith W.; Fourie C. M. T.; Botha S.; et al. The african prospective study on the early detection and identification of cardiovascular disease and hypertension (african-predict): Design, recruitment and initial examination. Eur. J. Prev Cardiol. 2019, 26, 458–470. 10.1177/2047487318822354. 30681377 PMC6423686

[ref23] Patro B. K.; Jeyashree K.; Gupta P. K. Kuppuswamy’s socioeconomic status scale 2010—the need for periodic revision. Indian J. Pediatr. 2012, 79, 395–396. 10.1007/s12098-011-0517-7. 21761123

[ref24] Stewart A.; Marfell-Jones M.; Olds T.; Ridder D. H. International society for advancement of kinanthropometry. International standards for anthropometric assessment; International Society for the Advancement of Kinanthropometry: Lower Hutt, New Zealand. 2011:50–53.

[ref25] Albalat A.; Bitsika V.; Zurbig P.; Siwy J.; Mullen W. High-resolution proteome/peptidome analysis of body fluids by capillary electrophoresis coupled with MS. Methods Mol. Biol. 2013, 984, 153–65. 10.1007/978-1-62703-296-4_12. 23386343

[ref26] R Core Team R: A Language and Environment for Statistical Computing; R Foundation for Statistical Computing: Vienna, AUhwR-po, (2018).

[ref27] Faul F.; Erdfelder E.; Lang A. G.; Buchner A. G*Power 3: A flexible statistical power analysis program for the social, behavioral, and biomedical sciences. Behav Res. Methods 2007, 39, 175–191. 10.3758/BF03193146. 17695343

[ref28] Szklarczyk D.; Gable A. L.; Nastou K. C.; Lyon D.; Kirsch R.; Pyysalo S.; Doncheva N. T.; Legeay M.; Fang T.; Bork P.; et al. The string database in 2021: Customizable protein-protein networks, and functional characterization of user-uploaded gene/measurement sets. Nucleic Acids Res. 2021, 49, D605–d612. 10.1093/nar/gkaa1074. 33237311 PMC7779004

[ref29] Vatner S. F.; Zhang J.; Vyzas C.; Mishra K.; Graham R. M.; Vatner D. E. Vascular Stiffness in Aging and Disease. Front. Physiol. 2021, 12, 762437 10.3389/fphys.2021.762437. 34950048 PMC8688960

[ref30] del Monte-Nieto G.; Fischer J. W.; Gorski D. J.; Harvey R. P.; Kovacic J. C. Basic biology of extracellular matrix in the cardiovascular system, part 1/4: Jacc focus seminar. J. Am. College Cardiol. 2020, 75, 2169–2188. 10.1016/j.jacc.2020.03.024. PMC7324287 32354384

[ref31] Xu J.; Shi G.-P. Vascular wall extracellular matrix proteins and vascular diseases. Biochim. Biophys. Acta, Mol. Basis Dis. 2014, 1842, 2106–2119. 10.1016/j.bbadis.2014.07.008. PMC4188798 25045854

[ref32] Zieman S. J.; Melenovsky V.; Kass D. A. Mechanisms, pathophysiology, and therapy of arterial stiffness. Arterioscler. Thromb. Vasc. Biol. 2005, 25, 932–943. 10.1161/01.ATV.0000160548.78317.29. 15731494

[ref33] Gros A.; Ollivier V.; Ho-Tin-NoÃ© B. Platelets in inflammation: regulation of leukocyte activities and vascular repair. Front. Immunol. 2015, 5, 678 10.3389/fimmu.2014.00678. 25610439 PMC4285099

[ref34] Bradley R. Gamma glutamyltransferase (GGT) as a biomarker of atherosclerosis. Biomarkers in cardiovascular disease; Springer: Dordrecht, Netherlands. 2016:673–702.

[ref35] Laronha H.; Caldeira J. Structure and function of human matrix metalloproteinases. Cells. 2020, 9, 1076 10.3390/cells9051076. 32357580 PMC7290392

[ref36] Peeters S. A.; Engelen L.; Buijs J.; Chaturvedi N.; Fuller J. H.; Jorsal A.; Parving H. H.; Tarnow L.; Theilade S.; Rossing P.; Schalkwijk C. G.; Stehouwer C. D. A.; et al. Circulating matrix metalloproteinases are associated with arterial stiffness in patients with type 1 diabetes: Pooled analysis of three cohort studies. Cardiovasc. Diabetol. 2017, 16, 139 10.1186/s12933-017-0620-9. 29070037 PMC5657128

[ref37] Yasmin; Wallace S.; McEniery C. M.; Dakham Z.; Pusalkar P.; Maki-Petaja K.; Ashby M. J.; Cockcroft J. R.; Wilkinson I. B. Matrix metalloproteinase-9 (MMP-9), MMP-2, and serum elastase activity are associated with systolic hypertension and arterial stiffness. Arterioscler., Thromb., Vasc. Biol. 2005, 25, 372 10.1161/01.ATV.0000151373.33830.41. 15556929

[ref38] Frantz C.; Stewart K. M.; Weaver V. M. The extracellular matrix at a glance. J. Cell Sci. 2010, 123, 4195–4200. 10.1242/jcs.023820. 21123617 PMC2995612

[ref39] Mels C. M.; Delles C.; Louw R.; Schutte A. E. Central systolic pressure and a nonessential amino acid metabolomics profile: the African Prospective study on the Early Detection and Identification of Cardiovascular disease and Hypertension. J. Hypertens. 2019, 37 (6), 1157–1166. 10.1097/HJH.0000000000002040. 30801385 PMC6513088

[ref40] Barallobre-Barreiro J.; Loeys B.; Mayr M.; Rienks M.; Verstraeten A.; Kovacic J. C. Extracellular matrix in vascular disease, part 2/4: Jacc focus seminar. J. Am. Coll. Cardiol. 2020, 75, 2189–2203. 10.1016/j.jacc.2020.03.018. 32354385

[ref41] Jiang S. Z.; Lu W.; Zong X. F.; Ruan H. Y.; Liu Y. Obesity and hypertension. Exp. Ther. Med. 2016, 12 (4), 2395–2399. 10.3892/etm.2016.3667. 27703502 PMC5038894

[ref42] Oh Y. S. Arterial stiffness and hypertension. Clin. Hypertens. 2018, 24, 17 10.1186/s40885-018-0102-8. 30519485 PMC6271566

