# Supplementary material for: Urinary Peptidomics and Pulse Wave Velocity: The African-PREDICT Study
Source: J Proteome Res. 2023 Sep 9;22(10):3282–9. doi: 10.1021/acs.jproteome.3c00347 (PMC10563154; doi:10.1021/acs.jproteome.3c00347)
Supplement: Supplementary file 1 — pr3c00347_si_001.pdf [file pr3c00347_si_001.pdf]

Urinary peptidomics and pulse wave velocity: the African-PREDICT study

Dalene de beer[1] (MHSc); Catharina MC Mels[1,2] (PhD); Aletta E Schutte[1,2,3] (PhD); Christian Delles[4] (MD); Sheon Mary[4] (PhD); William Mullen[4] (PhD); Harald Mischak[5] (PhD); Ruan Kruger[1,2] (PhD)

[1.] Hypertension in Africa Research Team (HART); North-West University (Potchefstroom Campus); Potchefstroom, South Africa, 2531
[2.] MRC Research Unit for Hypertension and Cardiovascular Disease, North-West University, Potchefstroom, South Africa, 2520
[3.] School of Population Health, University of New South Wales; The George Institute for Global Health, Sydney, Australia, NSW 2042
[4.] School of Cardiovascular and Metabolic Health, University of Glasgow, Glasgow, UK, G12 8QQ
[5.] Mosaiques Diagnostics GmbH, Hannover, Germany, D-30659

*Corresponding author:
Ruan Kruger, PhD
Hypertension in Africa Research Team (HART), North-West University
Private Bag X1290,
Potchefstroom, South Africa
Phone: +27 18 299 2904
Fax: +27 18 285 2432
Email: ruan.kruger@nwu.ac.za

Table of contents

Supplementary Table 1: Partially adjusted linear regression analyses between pulse wave velocity and urinary peptides in the total group

Supplementary Table 3: Multiple regression analysis of pulse wave analysis with identified peptides in the total group (all confounders included).

Supplementary Table 3: Interaction network of enriched Gene Ontology (GO) terms and pathway analysis of the identified urinary peptides.

Supplementary tables

Table S1: Partially adjusted linear regression analyses between pulse wave velocity and urinary peptides in the total group

		Pulse wave velocity (m/s)	
**Peptide ID**	**Peptide symbol**	**Total group (n=919)**	
e01100	COL1A1	r= -0.121	q= 0.02
e04169	COL1A1	r= -0.11	q= 0.02
e04199	COL2A1	r= 0.081	q= 0.04
e05200	COL5A3	r= 0.072	q= 0.05
e06213	COL3A1	r= 0.109	q= 0.02
e06961	COL3A1	r= -0.102	q= 0.02
e06978	COL1A1	r= -0.099	q= 0.03
e07944	COL1A1	r= 0.078	q= 0.03
e10072	COL3A1	r= 0.087	q= 0.03
e10351	COL4A2	r= 0.088	q= 0.04
e10445	COL1A1	r= 0.093	q= 0.02
e10876	COL3A1	r= -0.107	q= 0.02
e11265	COL1A2	r= 0.081	q= 0.04
e11879	COL1A2	r= 0.08	q= 0.04
e11972	COL1A1	r= 0.099	q= 0.02
e12851	COL1A1	r= 0.099	q= 0.02
e13253	COL3A1	r= 0.100	q= 0.02
e13707	COL3A1	r= 0.074	q= 0.04
e13850	COL1A1	r= 0.078	q= 0.05
e15467	COL1A2	r= 0.097	q= 0.02
e15863	COL1A1	r= 0.094	q= 0.02
e16170	COL1A1	r= 0.082	q= 0.03
e17596	COL1A1	r= 0.083	q= 0.04
e17599	PIGR	r= 0.093	q= 0.03
e17890	COL1A1	r= 0.115	q= 0.02
e19210	COL11A2	r= -0.089	q= 0.03
e19958	PIGR	r= 0.098	q= 0.02

Adjusted for mean arterial pressure.
Abbreviations: AU – arbitrary units. n – number of participants.

Table S2: Multiple regression analysis of pulse wave analysis with identified peptides in the total group (all confounders included).

		Pulse wave velocity	
		Total group (n=919)	
	Adj R2	Std b (±95 CI)	p-value
	0.39		
**e15863 (AU) (COL1A1)**		0.10 (0.04; 0.15)	**<0.001**
Age (years)		0.20 (0.15; 0.25)	**<0.001**
Body mass index (kg/m²)		-0.20 (-0.25; -0.14)	**<0.001**
sex (women/men)		0.28 (0.21; 0.34)	**<0.001**
Mean arterial pressure (mmHg)		0.37 (0.31; 0.43)	**<0.001**
γ-glutamyl transferase (U/l)		0.10 (0.04; 0.16)	**<0.001**
Heart rate (beats/min)		0.08 (0.02; 0.14)	**0.006**
	0.39		
**e15863 (AU) (COL1A1)**		0.08 (0.02; 0.13)	**0.007**
Age (years)		0.19 (0.13; 0.24)	**<0.001**
Body mass index (kg/m²)		-0.20 (-0.26; -0.14)	**<0.001**
sex (women/men)		0.28 (0.21; 0.34)	**<0.001**
Mean arterial pressure (mmHg)		0.37 (0.31; 0.43)	**<0.001**
γ-glutamyl transferase (U/l)		0.10 (0.04; 0.17)	**<0.001**
Heart rate (beats/min)		0.07 (0.01; 0.13)	**0.017**
	0.39		
**e17890 (AU) (COL1A1)**		0.08 (0.02; 0.14)	**0.011**
Age (years)		0.19 (0.13; 0.25)	**<0.001**
Body mass index (kg/m²)		-0.20 (-0.26; -0.14)	**<0.001**
sex (women/men)		0.27 (0.20; 0.34)	**<0.001**
Mean arterial pressure (mmHg)		0.37 (0.30; 0.43)	**<0.001**
γ-glutamyl transferase (U/l)		0.10 (0.04; 0.17)	**0.002**
Heart rate (beats/min)		0.07 (0.01; 0.14)	**0.023**
	0.39		
**e19958 (AU) (PIGR)**		0.07 (0.01; 0.12)	**0.019**
Age (years)		0.18 (0.12; 0.24)	**<0.001**
Body mass index (kg/m²)		-0.19 (-0.24; -0.13)	**<0.001**
sex (women/men)		0.29 (0.22; 0.35)	**<0.001**
Mean arterial pressure (mmHg)		0.37 (0.30; 0.43)	**<0.001**
γ-glutamyl transferase (U/l)		0.11 (0.05; 0.17)	**<0.001**
Low-density lipoprotein (mmol/L)		-0.06 (0.14; 0.002)	0.056
Heart rate (beats/min)		0.09 (0.03; 0.14)	**0.006**
	0.39		
**e12851 (AU) (COL1A1)**		0.07 (0.01; 0.13)	**0.027**
Age (years)		0.19 (0.13; 0.25)	**<0.001**
Body mass index (kg/m²)		-0.20 (-0.26; -0.13)	**<0.001**
sex (women/men)		0.27 (0.20; 0.34)	**<0.001**
Mean arterial pressure (mmHg)		0.37 (0.30; 0.43)	**<0.001**
γ-glutamyl transferase (U/l)		0.10 (0.03; 0.16)	**0.004**
Heart rate (beats/min)		0.07 (0.01; 0.14)	**0.032**
	0.39		
**e10445 (AU) (COL1A1)**		0.07 (0.004; 0.13)	**0.038**
Age (years)		0.21 (0.15; 0.27)	**<0.001**
Body mass index (kg/m²)		-0.20(-0.26; -0.13)	**<0.001**
sex (women/men)		0.26 (0.19; 0.33)	**<0.001**
Mean arterial pressure (mmHg)		0.36 (0.30; 0.43)	**<0.001**
γ-glutamyl transferase (U/l)		0.11 (0.04; 0.18)	**0.001**
Low-density lipoprotein (mmol/L)		-0.06 (-0.15; 0.002)	0.055
Heart rate (beats/min)		0.09 (0.02; 0.15)	**0.01**
	0.39		
**e11879 (AU) (COL1A2)**		0.06 (0.002; 0.12)	**0.044**
Age (years)		0.20 (0.14; 0.26)	**<0.001**
Body mass index (kg/m²)		-0.19 (-0.25; -0.13)	**<0.001**

sex (women/men)	0.28 (0.20; 0.35)	**<0.001**
Mean arterial pressure (mmHg)	0.36 (0.29; 0.43)	**<0.001**
γ-glutamyl transferase (U/l)	0.10 (0.03; 0.17)	**0.003**
Low-density lipoprotein (mmol/L)	-0.06 (-0.15; 0.01)	0.074
Heart rate (beats/min)	0.08 (0.02; 0.15)	**0.016**

Abbreviations: Adj – adjusted. Std β – standardised beta. AU – arbitrary units. n – number of participants.

Table S3: Interaction network of enriched Gene Ontology (GO) terms and pathway analysis of the identified urinary peptides. False discovery rate shows the significance of the enrichment shown as p-value corrected for multiple comparisons (Benjamini-Hochberg).

Molecular function (Gene Ontology)		
**GO-term**	**Description**	**False discovery rate**
GO:0030020	Extracellular matrix structural constituent conferring tensile strength	0.0110
GO:0048407	Platelet-derived growth factor binding	0.0040
**Reactome pathways**		
**Pathway**	**Description**	**False discovery rate**
HSA-430116	GP1b-IX-V activation signalling	0.0031
HSA-2214320	Anchoring fibril formation	0.0031
HSA-75892	Platelet Adhesion to exposed collagen	0.0031
HSA-2243919	Crosslinking of collagen fibrils	0.0031
HSA-3000480	Scavenging by Class A Receptors	0.0031
HSA-3000170	Syndecan interactions	0.0031
HSA-8874081	MET activates PTK2 signaling	0.0031
HSA-114604	GPVI-mediated activation cascade	0.0031
HSA-76009	Platelet Aggregation (Plug Formation)	0.0031
HSA-8948216	Collagen chain trimerization	0.0031
HSA-1442490	Collagen degradation	0.0049
HSA-3000178	ECM proteoglycans	0.0060
HSA-216083	Integrin cell surface interactions	0.0067
HSA-198933	Immunoregulatory interactions between a Lymphoid and a non-Lymphoid cell	0.0137
HSA-202733	Cell surface interactions at the vascular wall	0.0150
